# Explaining the self‐regulatory role of affect in identity theory: The role of self‐compassion

**DOI:** 10.1111/bjhp.12783

**Published:** 2025-02-01

**Authors:** Shaelyn M. Strachan, Sasha M. Kullman, Marko Dobrovolskyi, Vianney Z. Vega, Alexandra Yarema, Caity Patson

**Affiliations:** ^1^ Faculty of Kinesiology and Recreation Management University of Manitoba Winnipeg Manitoba Canada; ^2^ Department of Psychology University of Manitoba Winnipeg Manitoba Canada

**Keywords:** emotions, physical activity, self‐concept, self‐conscious emotions, self‐regulation

## Abstract

**Objectives:**

According to Stets and Burke's Identity Theory, people experience negative affect when their behaviour deviates from their identity standards, which drives the regulation of identity‐relevant behaviour. Guilt and shame represent unique forms of negative affect. Self‐compassion may influence guilt and shame responses about identity‐behaviour inconsistencies. Relative to exercise identity, we examined the associations between (1) guilt and shame, behavioural intentions, and perceptions of identity‐behaviour re‐alignment after an identity‐inconsistent situation and (2) whether self‐compassion moderates the relationship between these forms of negative affect and both behavioural intentions and identity‐behaviour re‐alignment.

**Design:**

Prospective, online, quantitative.

**Methods:**

*N* = 274 exercisers (*M*_age_ = 32.5 years, SD_age_ = 10.8 years, 50.2% women) who engaged in less exercise in the past week than their identity standard were recruited from Prolific.com. At baseline, self‐compassion, state and trait guilt and shame, and exercise intentions were measured. One week later, participants reported the extent to which their past week's exercise aligned with their identity standard (i.e., identity‐consistent perceptions).

**Results:**

Neither state shame nor guilt related to exercise intentions nor identity‐consistent perceptions. Self‐compassion moderated the relationship between state guilt and identity‐consistent perceptions (*b* = 2.524, SE = .975, *t* = 2.588, *p* = .010); state guilt was related to identity‐behaviour consistency when self‐compassion was high, but not when it was low. No other moderations were significant.

**Conclusions:**

This study adds nuance to Identity Theory and its propositions about negative affect and self‐regulation; self‐compassion may create the conditions necessary for negative affect to drive identity‐relevant behaviour as proposed by identity theory.

**Statement of contribution**

**What is known**

- Identity theory proposes negative affect as a driver of identity‐relevant behaviour.
- Contradictory research suggests negative affect thwarts behavioural regulation.
- Self‐compassion promotes adaptive responses to negative affect.

**What this study adds**

- When examined alone, the propositions of Identity Theory were not supported.
- Among people high in self‐compassion, state guilt was related to identity‐relevant behaviour.
- Self‐compassion may create the conditions necessary for state guilt to drive desired behaviours.

## INTRODUCTION

Identities are central to how people define themselves (Ryan & Deci, 2003), and identities have been studied from various perspectives, including cognitive (e.g., Markus, 1977) and developmental (Erikson, 1946). Proponents of the social‐psychological version of identity theory share the assumption that, through socialization, individuals develop a common understanding of social categories, which provides guidelines that make social behaviour predictable (Burke & Stets, 2009). Within this perspective, identities encompass the various roles (e.g., exerciser), groups (e.g., Métis), or unique personal attributes (e.g., extravert) that people occupy (Stets & Burke, 2003). Role identities, specifically, encompass the meanings associated with a given role (Burke & Stets, 2009), which entail what it means to hold that role identity. For example, the meanings associated with being a university student (role identity) may mean attending classes and devoting time to studying each day. Role identities and their associated meanings provide people with an internalized standard for behaviour whereby they work to behave in line with identity meanings (Burke & Stets, 2009). The stronger or more important an identity is to an individual, the more likely that person is to behave in line with identity meanings (Stets & Burke, 2003).

Identities are associated with identity‐consistent behaviour across a variety of domains, including environmental protection (Hinds & Sparks, 2008), prosocial behaviour (Bednall et al., 2013) and health behaviours (McCarthy et al., 2017), including exercise. A strong exercise identity is associated with exercise self‐regulation (Rhodes et al., 2016), and in‐turn, exercise frequency (Strachan et al., 2009), duration, intensity (Anderson & Cychosz, 1994), and maintenance (Strachan et al., 2016). In their meta‐analysis of 32 studies, Rhodes et al. (2016) describe exercise identity as one of the strongest psychological predictors of exercise (*r* = .44, 95% CI [.39–.48]).

### The identity control system

Stets and Burke's (2003) version of identity theory, with its emphasis on internal dynamics, is useful for understanding how role identities can lead to identity‐consistent behaviour through an identity control system (Stets & Burke, 2003). We, therefore, draw primarily on this version of identity theory within this paper. According to Stets and Burke (2003), when an identity is activated (e.g., an exerciser sees someone exercising), people consider the meanings of the current situation relative to their identity meanings, seeking a match. Alignment between situation meanings (e.g., ‘I did the exercise I planned to do today’) and identity meanings (e.g., ‘exercisers follow through on their exercise plans’) verifies the identity and produces positive emotions. A discrepancy between situation and identity meanings (e.g., ‘I did not exercise; exercisers follow through with their exercise plans’) threatens the identity, produces negative affect, and elicits a desire for behaviour change (e.g., ‘I will prioritize exercise tomorrow’) to align identity and situational meanings (Burke & Stets, 2015).

### Negative affect and the identity control system

Affect is central within identity theory and its identity control system; when there is a mismatch between situational and identity meanings, negative affect should motivate change. Some studies support this role of negative affect within Identity Theory. Yang and Zhang (2023) found that occupational identity challenges were associated with negative affect, which was positively related to identity‐confirming behaviours. Other studies have only examined behavioural intentions within identity theory, rather than actual behaviours. Strachan and Brawley (2008) demonstrated that negative affect related to identity‐disconfirming events was associated WITH THE INTENTION TO verify ones' healthy‐eater identity. Similarly, research on exercise identity using hypothetical situations (Strachan & Brawley, 2008), real‐life discrepancies (Strachan et al., 2009), and experiments (Strachan et al., 2018) have found that exercisers faced with an identity‐disconfirming situation react with negative affect and intentions for identity‐behaviour consistency. However, little research examines whether these negative feelings about an identity challenge translate into future perceptions of identity‐behaviour consistency.

Other research shows an inconsistent relationship between negative affect and identity‐relevant behaviours, calling into question this tenet of identity theory. Miller and Kalkhoff (2020) exposed students to a manipulation where they received disconfirming feedback about their student identities (e.g., students received feedback that they were ‘non‐studious’ after indicating they were ‘very studious’ on a questionnaire). Next, students completed measures assessing negative affect and future intentions to engage in identity‐relevant behaviours (e.g., studying). Contrary to identity theory, students who were faced with more identity‐disconfirming feedback experienced more negative affect, but higher levels of negative affect did not translate into stronger behavioural intentions for studying (Miller & Kalkhoff, 2020). Within the exercise context, limited research exists to explore this relationship. In their prospective, observational study, Meade et al. (2020) found that exercisers experienced negative affect in the form of guilt and shame after a real‐life missed exercise session (i.e., identity‐inconsistent behaviour). Contrary to identity theory's predictions, guilt negatively predicted exercise intentions, and neither guilt nor shame predicted the likelihood of engaging in planned exercise (Meade et al., 2020). These mixed findings highlight the need for additional research.

### Negative affect and self‐regulation

Identity theory's notion that negative affect motivates and promotes goal‐directed behaviour has been criticized and is at odds with findings within the self‐regulatory literature. However, it should be acknowledged that research on self‐regulation generally considers negative affect broadly, whereas identity theory research contextualizes negative affect within an identity challenge. Stets and Burke (2003) borrow from Carver andScheier's (1981) Control TheoPry to explain how negative affect motivates behavioural regulation; when output falls below a standard, negative affect signals a disturbance and steps are taken to return output to the standard. Ryan and Deci (1999) are critical of such a simplistic approach to self‐regulation, arguing that it fails to incorporate human needs or consider the source of goal‐directed behaviour (i.e., internal or external to the self; Ryan & Deci, 1999). Further, self‐regulation theorists argue that negative affect can undermine (rather than motivate) successful goal pursuit (Tice et al., 2001). For instance, negative affect has been shown to impair decision‐making (Bruyneel et al., 2009) and self‐regulation (Townsend & Liu, 2012), direct attention away from goals (Schmeichel, 2007), decrease effort and engagement (Capa & Audiffren, 2009), promote procrastination (Sirois, 2014), and is inversely related to state persistence (Steinberg & Williams, 2013). Indeed, negative affect was inversely associated with health‐promoting behaviours (*b* = −.214 to −.370, *p* < .05) among four of eight samples (for which affect data was available) as reported in a larger meta‐analysis involving 15 independent samples (*N* = 3252; Sirois et al., 2015). Taken together. these studies offer an alternative viewpoint about the role of negative affect in motivated behaviour.

### Guilt and shame

A conversation about the role of negative affect in motivating behaviour should also consider the *type* of negative affect experienced. Guilt and shame are self‐conscious emotions experienced following departure from ones' goals (Tangney, 2003), such as when people fail to behave consistently with an endorsed identity. These emotions influence behaviours uniquely (Tracy & Robins, 2004). Shame involves blaming the global self for failure (e.g., ‘I am ashamed of myself for missing another exercise session. I am so lazy!’), viewing the failure as uncontrollable (e.g., ‘I will never be consistent’), and disengagement from the task (e.g., ‘I'd better not waste my time’; Lewis, 2019; Tracy & Robins, 2006). Therefore, when a person behaves at odds with their identity and feels shame, they may become overwhelmed and unmotivated such that they disengage from the behaviour. Research supports this idea; following a face‐saving failure, shame‐prone students tried less on a cognitive task than their less shameful counterparts (Thompson et al., 2004). Conversely, guilt is a negative evaluation of ones' specific behaviour, rather than ones' global self (e.g., ‘I feel bad for not running today’). Guilt involves viewing failure as controllable (e.g., ‘I can try again’), and guilt may motivate adaptive behaviour (e.g., ‘I'll run tomorrow’; Tracy & Robins, 2006). If a person behaves at odds with their identity and feels guilt, the guilt may motivate change. For example, Burmeister et al. (2019) found that engaging in deceptive behaviour (i.e., playing dumb) towards coworkers was linked to guilt and shame; however, following this behaviour, guilt was positively related to compensatory action, while shame was negatively related to this outcome. Therefore, although research, including identity theory research, often focuses on global negative affect (e.g., Strachan & Brawley et al., 2008, 2009), the distinction between guilt and shame makes it important to distinguish between them in research (Meade et al., 2020).

### Self‐compassion

A person's ability to respond to feelings of guilt and shame with *self‐compassion* may add nuance to the relationship of negative affect with both behavioural intentions and engagement in identity‐consistent behaviours when an identity is threatened. Self‐compassion is a psychological resource involving the extension of care and understanding to oneself in situations of challenge or failure (self‐kindness), viewing suffering as a shared human experience (common humanity), and holding suffering in balanced awareness rather than overidentifying with or disconnecting from it (mindfulness; Neff, 2003a; Germer & Neff, 2018).

Self‐compassion is associated with the experience of fewer negative emotions in the face of challenges (Neff, 2003a) including guilt and shame (e.g., Etemadi Shamsababdi & Dehshiri, 2024; Sirois et al., 2019). In the exercise context, self‐compassion was associated with less guilt about taking time away from ones' child or children to engage in health behaviours, including exercise, among mothers of young children (Miller et al., ). Limited research has examined self‐compassion, guilt, and shame in the context of an exercise identity threat. Kullman et al. (2021) found that self‐compassion was negatively associated with shame among mothers who identified as exercisers and no longer met their pre‐parenthood exercise identity standards. However, self‐compassion was unrelated to guilt in this situation. Self‐compassion's association with fewer negative emotions (Woods & Proeve, 2014) is particularly beneficial in the case of shame, given shame's association with goal disengagement (Lewis, 2019). Little research has explored the interplay between self‐compassion and guilt and shame as separate emotions, indicating an area needing further inquiry (Woods & Proeve, 2014). Further, more research is needed to understand the association between self‐compassion with both guilt and shame in the context of identity theory.

In addition to its purported association with fewer negative emotions, self‐compassion can also *mitigate* the effect of the negative emotions people experience in the face of a challenge, by helping them tolerate (Diedrich et al., 2016) and regulate these emotions (Miyagawa et al., 2018; Semenchuk, 2018). When people experience setbacks, self‐compassion creates an emotionally safe internal environment where people can consider their failure without developing negative self‐evaluations (Leary et al., 2007). Given that self‐compassion is associated with a desire to improve (Breines & Chen, 2012) and wanting what is best for oneself (Neff, 2003a), self‐compassion may allow people to *embrace* guilt (Kullman et al., 2021) so that it motivates self‐improvement (Brienes & Chen, 2012). Specifically, self‐compassionate people should be mindful of their guilt, recognize guilt as a signal for behaviour change, and experience guilt along with self‐kindness and a sense of common humanity. A self‐compassionate approach should not eliminate feelings of guilt altogether, but should lessen or change the impact of these feelings (Miller & Strachan, 2020) such that guilt can motivate action that is in peoples' best interest. Self‐compassion, when in place, may explain how guilt experienced by people who have behaved at odds with their identity meanings, can lead to intentions for, and engagement in identity‐consistent exercise, as outlined by identity theory.

### The present study

The first purpose of this study is to examine the Identity Theory proposition (Stets & Burke, 2003) that negative affect about an identity‐disconfirming situation relates positively to intentions to re‐engage in identity‐confirming behaviours, and subsequent perceptions of identity‐behaviour re‐alignment. It is important that we examine these simple effects given the dearth of research that has examined these identity‐theory predictions. We will follow recommendations to examine the differential effects of different emotions, particularly guilt and shame (Tracy & Robins, 2004). Our hypotheses were established apriori and are listed as follows: Hypothesis (1) Given that shame can promote disengagement from identity‐relevant behaviour (Lewis, 2019), shame will be negatively related to intentions for, and perceptions of having engaged in identity‐consistent behaviour. Hypothesis (2) Though guilt can motivate reparative action (Tracy & Robins, 2004), we hypothesize that it will be unrelated to intentions for, and perceptions of having engaged in identity‐consistent behaviour. We reason that guilt will only be associated with motivation and engagement in identity‐consistent behaviour when people are also self‐compassionate.

Therefore, a second purpose of this study is to test the moderating role of self‐compassion within the identity control System (Stets & Burke, 2003). Hypothesis (3) Self‐compassion will moderate the relationship between state guilt and intentions for, and perceptions of having engaged in identity‐consistent behaviour. Specifically, state guilt will be positively associated with these outcomes when self‐compassion is high, but not when self‐compassion is low. In other words, the propositions of the identity control system (Stets & Burke, 2003) will be fulfilled only in those who are high in self‐compassion. Hypothesis (4) Given that shame is not constructive for self‐regulation, we hypothesize no such moderated relationships will emerge for shame.

## MATERIALS AND METHODS

This prospective, online study received ethical approval from the human ethics board at a large Canadian university. Study measures were housed on SurveyMonkey. The following parameters were entered into G*Power 3.1 software to calculate sample size: 80% power, two‐sided 5% significance level, and one group with six predictors (Hulley et al., 2013). Regarding anticipated effect size, we drew from the sizes of other theoretically relevant relationships to inform our power calculation. We conservatively estimated a small‐medium effect size given that past research has shown both small (*η* ^2^ = .006; Strachan & Brawley, 2008) and medium‐sized (*η* ^2^ = .133; Strachan et al., 2018) relationships between exercise identity and negative affect in the context of an exercise identity threat. Furthermore, a small‐to‐medium‐sized relationship has been identified between self‐compassion and health behaviour intentions (*r* = .26; Sirois, 2015). Drawing from these studies, we estimated that *N* = 274 participants would provide sufficient power for the planned analyses (Hulley et al., 2013).

### Participant recruitment and eligibility screening procedures

Participants were recruited through the online crowdsourcing platform, Prolific.com. Prolific.com users over 18 years of age, fluent in English, who indicated ‘health and fitness’ as a hobby and reported engaging in >150 min of exercise per week on their profile were invited to consent and complete a survey which assessed additional eligibility criteria as described below. Participants were eligible for this study if they scored above the scale mean on exercise identity (>3.5‐points), could safely engage in exercise, and reported that they were currently experiencing an exercise identity‐disconfirming situation. Eligibility responses were screened within 1 day of submission.

#### Eligibility measures

##### Exercise identity

The reliable and validated Exercise Identity Scale (Anderson & Cychosz, 1994) was used to measure exercise identity. This scale contains 9 items answered on a 7‐point Likert scale (1 = *strongly disagree* to 7 = *strongly agree*). A sample item is: ‘physical activity is a central factor to my self‐concept’. The mean score of all scale items represents exercise identity. This scale had good internal consistency (*α* = .866).

##### Exercise safety

Participants responded to two questions assessing whether they could safely engage in exercise. First, they indicated whether they currently had injuries that prohibited exercise (*yes/no*). Next, they indicated whether a healthcare provider had contraindicated their exercise (*yes/no*). If participants responded ‘no’ to both of these questions, they were deemed safe to engage in exercise.

##### Identity‐disconfirming situation

We used the following procedure to identify participants who were experiencing an identity‐disconfirming situation and increased the salience of this discrepancy for those to which it applied. First, we prompted participants to reflect on the behavioural meaning of their exercise identity by reading the following script:

When we see ourselves holding a given role (e.g., parent, friend), there are things we need to do in order to live “true to” or in line with that role. For example, if you see yourself as a “good friend” you may need to touch base with your friend every week or return your friend's text messages promptly. You are participating in this study because you see yourself as an exerciser. As with the above example, there would be things you need to do to say you are an “exerciser”. We want to get a sense of what you need to do to see yourself as an exerciser. To help us understand this, please use the prompts below to answer the following question: How much exercise do you have to do in a typical week for your behaviour to be consistent with your personal view of yourself as an exerciser?

After reading this script, participants described the behavioural standards required to confirm their exerciser identities by completing the reliable and valid Godin Leisure Time Exercise Questionnaire (GLTEQ; Godin & Shephard, 1985). Specifically, participants reported the frequency and duration of mild, moderate, and strenuous exercise they would have to do in a week to behave in line with their exerciser identity. We reasoned that this procedure made participants' personal exercise identity salient.

Next, participants were prompted to reflect on the amount of exercise they had completed in the past week by reading the following statement:

We want to understand the amount of physical activity, if any, that you did in the past week. Please do your best to answer as precisely and honestly as possible, and do not simply respond with what you think we ‘would like to hear’. Don't worry if you have not done as much physical activity as you wish you had.

After reading this statement, participants completed the GLTEQ a second time to report the amount of mild, moderate, and vigorous exercise they had completed in the past week. Finally, participants responded with ‘yes’ or ‘no’ to the following question *‘In thinking about the exercise you did in a recent typical week (that you just reported), is this behaviour consistent with the amount of exercise you need to do to see yourself as an “exerciser”*?’.

To meet the study eligibility criteria, participants must have indicated they were experiencing an identity‐disconfirming situation by responding with ‘no’ to this question. In addition, participants rated how consistent their past week's exercise was with their identity standard on a scale ranging from 0% (*not at all consistent with my exercise identity*) to 100% (*completely consistent with my exercise identity*). This percentage score was used for descriptive purposes only. We successfully piloted and applied this procedure for assessing exercise‐related identity‐behaviour inconsistency in previous research (Kullman et al., 2021). In the present study, we piloted the script on an undergraduate student who identified as an exerciser to confirm it was understandable. Furthermore, comprehension check items such as ‘*do you understand the instructions?*’ (*yes/no*) were included after each written prompt.

### Study procedures

Eligible participants were invited to complete two surveys assessing the study outcomes on Prolific.com. The Time 1 survey was administered to participants within 1 day of completing the eligibility measures, and the Time 2 survey was administered 1 week after participants completed the Time 1 survey. Participants were required to complete both surveys within 2 days of receiving them. In line with Prolific.com's ethical compensation guidelines, participants received $2.15 CAD for completing the eligibility survey, $6.00 CAD for completing the Time 1 Survey, and $2.15 CAD for completing the Time 2 Survey. Items which asked participants to type specific words or phrases into text boxes were distributed throughout the survey to ensure careful responding. Participants were debriefed via a written message administered through Prolific.com after the study's conclusion.

#### Time 1 measures

##### Demographics

Participants reported their age, education, geographic location, ethnicity, gender identity, and marital status.

##### Self‐compassion

The reliable and validated 26‐Item Self‐Compassion Scale (Neff, 2003b) has six subscales which assess the positive and negative aspects of self‐compassion (self‐kindness, mindfulness, common humanity, self‐criticism, over‐identification, and isolation). A sample item is, ‘*When I'm going through a very hard time, I give myself the caring and tenderness I need*’. Responses are scored on a 5‐point Likert scale (1 = *almost never* to 5 = *almost always*). To determine total self‐compassion, negative items are reverse‐scored, and a grand mean is calculated. This scale had good internal consistency (*α* = .922).

##### State guilt and shame

The reliable and validated State Shame and Guilt Scale (Marschall et al., 1994) uses five items to assess state shame (e.g., ‘*I feel like I am a bad person*’) and five items to assess state guilt (e.g., ‘*I feel remorse, regret*’). Participants were asked to reflect on their exercise identity‐disconfirming situation while responding to the items using a 5‐point Likert scale (1 = *I don't feel this way at all* to 5 = *I feel this way very strongly*). State guilt and shame scores were created by summing responses to both subscales, respectively.^1^ Both state guilt (*α* = .790) and state shame (*α* = .861) subscales had good internal consistency.

##### Trait guilt and shame

The reliable and validated Test of Self‐Conscious Affect (Tangney et al., 2000) uses 16 scenarios to measure guilt and shame‐proneness. An example scenario is, ‘ *You make plans to meet a friend for lunch. At 5 o'clock, you realize you stood your friend up* ’. Participants report how likely they are to behave in certain ways in each scenario using a 5‐point Likert scale (1 = *not likely* to 5 = *very likely*). A sample response is ‘*You would think: I am inconsiderate’*. Trait guilt and shame were calculated by summing each response relating to guilt and shame, respectively. Both trait guilt (*α* = .738) and trait shame (*α* = .818) subscales had good internal consistency.

##### Exercise intentions

Participants responded to the statement ‘Do you intend to increase your current activity level?’ using a 7‐point Likert scale (1 = *strongly disagree* to 7 = *strongly agree*). Higher scores indicated stronger exercise intentions. This method of assessing exercise intentions has been used in previous similar research (Kullman et al., 2021; Milne et al., 2008).

#### Time 2 measures

##### Perceptions of identity‐behaviour consistency

Using a similar procedure to that employed during eligibility screening, we assessed whether participants were still experiencing an identity‐disconfirming situation at Time 2. Participants first reported the frequency and duration of mild, moderate, and vigorous physical activities they had completed in the past week using the GLTEQ (Godin & Shephard, 1985). Participants then responded to the following question:

In thinking about the exercise you did the past week, how consistent was your exercise with the amount of exercise you need to do to see yourself as an exerciser? Please express this as a percentage. For example, if you reported that you need to exercise at a moderate intensity 3 times per week to see yourself as an exerciser, but *in the past week you* only exercised twice, your answer would be 75%.

Participants responded to this question using a scale ranging from 0% (*not at all consistent with my exercise identity*) to 100% (*completely consistent with my exercise identity*). This percentage score was used as a main outcome variable called ‘identity‐consistent perceptions’. Comprehension check items were once again included after each written prompt.

### Data management and analysis

Data cleaning followed the recommendations of Tabachnick and Fidell (2014) and Pallant (2020). The analyses were conducted using IBM SPSS version 29. The data met the assumptions of linearity, independence, normality, and homoscedasticity (Pallant, 2020). Univariate outliers were addressed using Winsorization (Tabachnick & Fidell, 2014). Age and gender were controlled for in each statistical analysis. In all analyses examining state shame and state guilt, we controlled for trait shame and trait guilt, respectively. All regression analyses were conducted using stepwise entry whereby control variables were entered in the first blocks, and the predictor variable in the final block.

To test Hypotheses 1 and 2, we ran four separate hierarchical regression analyses relating state shame and state guilt to exercise intentions and Time 2 identity‐consistent perceptions. To test Hypothesis 3, we used Hayes PROCESS macro version 4.2 for SPSS (Hayes et al., 2017). Using Model 1, state guilt was entered as the predictor variable, self‐compassion as the moderator, and trait guilt, age, and gender were entered as covariates simultaneously. Exercise intentions and Time 2 identity‐consistent perceptions were then individually entered as outcomes in two separate moderation analyses. To test Hypothesis 4, this same process was repeated using state shame as the predictor variable and trait shame, age, and gender as covariates. Continuous variables were mean‐centred. We examined the effects of state guilt and shame on the outcomes at three levels of self‐compassion: the mean, plus and minus one standard deviation from the mean. The Johnson‐Neyman technique was used to determine the region of significance for any significant models.

## RESULTS

Of the participants who completed the eligibility screening process, 53.3% met the eligibility criteria and were granted access to the Time 1 survey. Participants (*N* = 274) were an average of 32.5 years old (SD = 10.8 years; range = 18–65 years), 66.1% identified as White, and 50.2% identified as women (Table S1). All participants responded affirmatively to the comprehension and attention check items throughout the surveys. The sample scored in a moderate range on self‐compassion (*M* = 2.97 points, SD = .64 points) and high on exercise identity (*M* = 5.71 points, SD = .78 points). At Time 1, participants reported engaging in *M* = 50.1% (SD = 21.9%) of the exercise they required to behave in line with their exercise identity. This identity‐inconsistent situation allowed us to examine the relationship between negative affect with both exercise intentions and subsequent perceptions of identity‐consistent behaviour at Time 2, as well as the moderating role of self‐compassion. Table 1 shows a correlation matrix of all key variables. Descriptive statistics are provided in Table S2.

**TABLE 1:** Correlations between all study variables.

		1	2	3	4	5	6	7	8	9
1	Age		.086	.123*	−.240**	−.196**	−.098	.053	.005	.097
2	Exercise Identity			.109	.076	.071	−.237**	.107	.062	.163**
3	Self‐compassion				−.175**	−.217**	−.514**	−.043	.026	.174**
4	State Shame					.697**	.209**	.010	.099	−.009
5	State Guilt						.237**	−.010	.101	−.046
6	Trait Shame							.408**	−.031	−.129*
7	Trait Guilt								.114	−.033
8	PA intentions									−.086
9	Time 2 identity‐ consistent perceptions									

*p < .05, **p < .01.

Regarding the first study purpose, Hypothesis 1 was not supported; state shame was unrelated to exercise intentions (*b* = .052, SE = .029, 95% CI [−.005, .109], *p* = .075) and Time 2 identity‐consistent perceptions (*b* = .387, SE = .664, 95% CI [−.921, 1.695], *p* = .561). Hypothesis 2 was supported; state guilt was unrelated to exercise intentions (*b* = .052, SE = .029, 95% CI [−.005, .109], *p* = .073) and Time 2 identity‐consistent perceptions (*b* = −.124, SE = .670, 95% CI [−1.443, 1.195], *p* = .854; Tables S3–S6).

**FIGURE 1:**
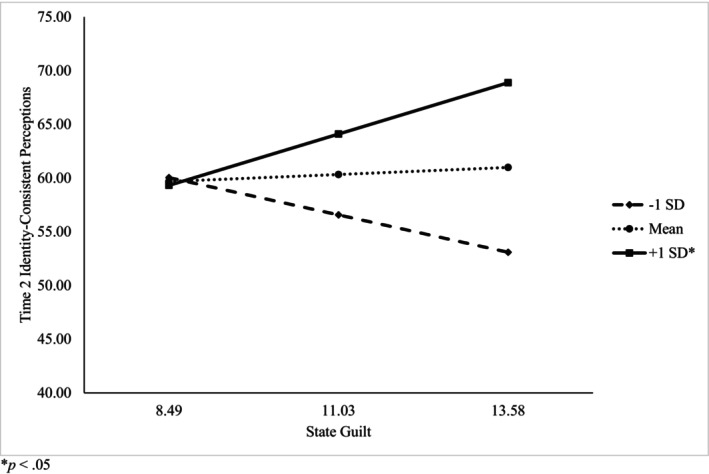
Moderating effect of self‐compassion on the relationship between state guilt and Time 2 identity‐consistent perceptions.

Regarding the second study's purpose, Hypothesis 3 was partially supported. The moderation model using state guilt as a predictor, self‐compassion as a moderator, and Time 2 identity‐consistent perceptions as the outcome showed a good fit to the data, (*F* [8, 251] = 2.750, *p* = .006, *R* ^2^ = .081). The interaction between state guilt and self‐compassion was significant (*b* = 2.524, SE = .975, 95% CI [.603, 4.445], *t*[251] = 2.588, *p* < .0102) indicating that self‐compassion moderated the relationship between state guilt and identity‐consistent perceptions (Figure 1, Table 2). The addition of the interaction contributed significantly to the model (*F*[1, 251] = 6.698, *R* ^2^‐change = .025, *p* = .0102). Simple slope analysis showed that at one standard deviation below the mean self‐compassion score, and at the mean, state guilt did not predict perceptions of identity‐behaviour consistency (*p* > .05). However, at one standard deviation above the mean self‐compassion score, state guilt significantly predicted perceptions of identity‐behaviour consistency (*b* = 1.879, *SE* = .929, 95% CI [.050, 3.708], *t*[251] = 2.024, *p* = .0441; Table 3). Therefore, for people high in self‐compassion, state guilt predicted greater perceptions of identity‐behaviour consistency 1 week following an identity‐disconfirming situation. The Johnson‐Neyman technique was used to probe the region of significance and demonstrated that at self‐compassion scores of 3.57 and greater, state guilt was significantly related to identity‐behaviour consistency. However, self‐compassion did not moderate the relationship between state guilt and exercise intentions (*b* = −.028, SE = .043, 95% CI [−.112, .057], *t*[251] = −.640, *p* = .523; Table S7). Finally, Hypothesis 4 was supported; self‐compassion did not moderate the relationship between state shame and exercise intentions (*b* = −.027, SE = .045, 95% CI [−.115, .061], *t*[259] = −.609, *p* = .543) or Time 2 identity‐consistent perceptions (*b* = 1.166, SE = 1.012, 95% CI [−.827, 3.159], *t*[259] = 1.152, *p* = .250; Tables S8 and S9).

**TABLE 2:** Interaction effect of self‐compassion and state guilt on Time 2 identity‐consistent perceptions.

Variable	*b*	95% CI for *b*	SE of *b*	*t*	*p*
Constant	78.5210	−10.2078 to 167.2498	45.0523	1.7429	.0826
State Guilt	−7.2472	−13.0691 to −1.4253	2.9561	−2.4516	.0149
Self‐Compassion	−21.9941	−44.0921 to .1040	11.2203	−1.9602	.0511
State Guilt*Self‐Compassion	2.5242	.6033 to 4.4452	.9754	2.5879	.0102
Trait Guilt	.0617	−.3959 to .5193	.2323	.2656	.7908
Age	.3075	−.0028 to .6179	.1576	1.9515	.0521
Gender (Man)	33.6792	−18.0484 to 85.4067	26.2648	1.2823	.2009
Gender (Woman)	27.1547	−24.6274 to 78.9369	26.2925	1.0328	.3027

**TABLE 3:** Simple slope estimates for the interaction effect of self‐compassion and state guilt on Time 2 identity‐consistent perceptions.

Variable	*b*	95% CI for *b*	SE of *b*	t	*p*
Low self‐compassion (−1 SD; SC = 2.330)	−1.3658	−3.1416 to .4101	.9017	−1.5147	.1311
Average self‐compassion (SC = 2.973)	.2565	−1.0566 to 1.5697	.6668	.3848	.7007
High self‐compassion (+1 SD; SC = 3.6154)	1.8789	.0503 to 3.7075	.9285	2.0236	.0441

Abbreviation: SC, self‐compassion.

## DISCUSSION

This study examined the identity theory proposition that negative affect about an identity‐disconfirming situation should positively relate to identity‐relevant intentions and subsequent perceptions of identity‐behaviour re‐alignment (Stets & Burke, 2003). We also examined the moderating role of self‐compassion within this relationship. We used ‘exercise identity’ as the context in which to frame this study, and we examined guilt and shame as two distinct facets of negative affect. When considering the direct relationships from negative affect (i.e., guilt and shame) to identity‐relevant outcomes, identity theory was not supported. We found that self‐compassion significantly moderated the relationship between guilt and perceptions of identity‐behaviour consistency. Overall, the role of negative affect within identity theory may hold true among people who are more self‐compassionate.

### Negative affect and the identity control system

The few studies examining Identity Theory's propositions around the role of negative affect in the regulation of identity‐relevant behaviours are mixed (Meade et al., 2020; Yang & Zhang, 2023), and other research suggests that negative affect may undermine self‐regulation (e.g., Sirois et al., 2015). Our findings did not align with Identity Theory, nor did they suggest that guilt or shame were related to identity‐relevant behaviour. Indeed, neither guilt nor shame about behaving inconsistently with ones' exercise identity were related to greater exercise intentions or perceptions of identity‐behaviour consistency. Acknowledging the dearth of research that examines affect within an identity challenge, we considered research that examines general affect and physical activity, finding no clear conclusion to be drawn. For example, research supports a negative (e.g., Sirois et al., 2015), positive (Brunet & Sabiston, 2011; Edmunds et al., 2006) and null relationship (e.g., Meade et al., 2020) between negative affect or related concepts (e.g., shame, guilt) and physical activity. These mixed findings hold among behaviours other than exercise (e.g., employment and education; Burmeister et al., 2019; Thompson et al., 2004). It is likely that whether negative affect facilitates, undermines, or is unrelated to health behaviours (that people may or may not identify with) depends on moderating factors.

### Guilt and shame

Guilt and shame are self‐conscious emotions that fall under the umbrella of ‘negative affect’ which may differently influence behaviours and cognitions. We improved upon past research by examining these two constructs independently. Shame involves self‐blame, viewing failure as uncontrollable, and disengagement (Tracy & Robins, 2004). We found that shame was unrelated to exercise intentions and perceptions of identity‐behaviour consistency. One explanation is that participants did not feel shameful about their identity‐discrepant situation. On our measure of state shame, the sample mean was only 11.93 points (SD = 2.60 points) out of 25 points. Although participants likely valued exercise, the consequences of skipping a few exercise sessions may not have been harmful enough to elicit shame. Shame was also unrelated to exercise in other research (Meade et al., 2020). Perhaps shame is more salient when the consequences of identity‐behaviour inconsistency cause harm to oneself or others. For example, someone who identifies as a ‘gentle parent’ may feel shame after losing their temper with their child. Researchers should explore Identity Theory propositions in the context of other identities to see if these relationships hold true.

Guilt is experienced about behaviours we believe we can change and may motivate reparative action (Tracy & Robins, 2004). Given inconsistent findings on guilt and behaviour in other studies (e.g., Meade et al., 2020; Miller & Kalkhoff, 2020), we reasoned that guilt alone would not relate to identity‐consistent outcomes; this hypothesis was confirmed. Notably, participants experienced relatively low levels of guilt (11.03 points out of 25 points, SD = 2.49). However, guilt may be more applicable to the exercise context, as exercise is a morally‐laden behaviour in Western society (Zanker & Gard, 2008) and exercisers experience guilt in relation to exercise lapses (Flora et al., 2012). Though guilt alone may be insufficient to impact identity‐relevant outcomes, we reasoned that when people respond to their guilt with self‐compassion, guilt may be a motivator for identity‐relevant reparative action.

### The moderating role of self‐compassion in the identity control system

Often, when the relationship between two variables is inconsistent across studies, as is the case with the relationship between negative affect and behavioural regulation, there may be a moderator at play (Memon et al., 2019). We reasoned that an examination of the potential moderating role of self‐compassion may provide more nuance to the relationship between negative affect and identity‐related behavioural regulation. We found that self‐compassion significantly moderated the relationship between guilt and perceptions of identity‐behaviour consistency. Specifically, among people high in self‐compassion, state guilt was related to greater perceptions of identity‐behaviour consistency, whereas no relationship existed between state guilt and this outcome among those lower in self‐compassion. Our findings suggest that self‐compassionate exercisers may be better able to re‐align their exercise behaviour with their identity standards after experiencing an identity‐inconsistent situation. Self‐compassion should allow people to view guilt about their identity challenge in a balanced manner without becoming overwhelmed by negative affect (i.e., mindfulness), create an inner dialogue of supportive coaching rather than harsh self‐criticism (i.e., self‐kindness), and normalize failure (i.e., common humanity). Therefore, self‐compassion should protect against the self‐regulatory downfalls of negative affect and create an environment where guilt can motivate reparative action. The finding that self‐compassion may create a context where guilt can be motivating is important because behaving in a way that aligns with ones' identity can affirm, strengthen, and reinforce that identity (Stets & Burke, 2023). If someone is chronically unable to meet their identity standard, they may abandon their identity (Stets & Burke, 2023). Therefore, if self‐compassion makes it more likely that one succeeds at confirming their identity after a deviation, a possibility based on our findings, self‐compassion may be a tool that can help people navigate identity challenges.

The finding that self‐compassion moderates the relationship between guilt and perceptions of identity‐behaviour consistency presents avenues for future research. First, self‐compassion may work synergistically with other morally‐laden health behavioural identities to encourage identity‐behaviour consistency, such as being a healthy eater or a non‐smoker, and this possibility could be tested. If our findings are replicated, self‐compassion training (e.g., Germer & Neff, 2018) could be coupled with interventions designed to strengthen desirable identities, including those targeting exercise (e.g., Cooke et al., 2020; Hollman et al., 2022), as a constructive way to overcome identity‐related challenges. For example, techniques to build identity may focus on having people reflect on their core values (e.g., family, independence) and how the identity‐relevant behaviour aligns with those values (e.g., engaging in exercise sets a good example for and provides an opportunity to spend time with family). Further, people may be prompted to clearly define and outline their identity standard (e.g., for me, being an exerciser means going for a 30‐min walk, 5 days per week) so that they have a clearly defined, salient identity standard to pursue. Along with these identity‐building strategies, people could be introduced to self‐compassion and taught how to apply it to the pursuit of identity‐relevant behaviour. People could be taught to apply self‐compassion when they fall short of their identity standard through activities such as writing a compassionate letter to oneself or noticing self‐critical self‐talk and replacing it with self‐compassionate talk (Germer & Neff, 2018). Augmenting identity‐building interventions with self‐compassion training may be suited to populations for whom adopting a beneficial identity may be especially challenging. Two examples include people living with, or at‐risk for, a chronic condition, and new parents; both these populations struggle to be active (Bellows‐Rieken & Rhodes et al., 2008; Semenchuk et al., 2022) and stand to benefit from exercise. Their development of an exercise identity would facilitate exercise behaviour (Rhodes et al., 2016). Self‐compassion is advantageous in the face of exercise‐related challenges (Semenchuk et al., 2018) and could increase the chances that an identity‐building intervention is successful with these populations. Interventions of this nature could be tested in the future.

Self‐compassion did not moderate the relationship between guilt and exercise intentions. This was surprising because the guilt that exercisers felt was related to their perceptions of identity‐behaviour consistency; one would think that this behaviour would be accompanied by an intention. Our single‐item intention measure may be too simplistic to capture exercise intentions. Though this measure has been used in past research (Kullman et al., 2021), other more comprehensive measures of exercise intentions may be more useful in future research. Finally, we found that self‐compassion did not moderate the relationship between shame and either of our outcomes. This finding was expected, given that shame promotes disengagement (Lewis, 2019) and may not be helpful for self‐regulation.

### Strengths and limitations

This research adds to identity theory by suggesting self‐compassion may explain the conditions under which predictions about negative affect are most likely to be supported. This study also adds to research on exercise identity, where only a handful of studies have examined the identity control system (Strachan & Whaley, 2013). Methodological strengths include our prospective design and external validity; we recruited people who were experiencing a real‐life identity‐discrepant situation and examined their behaviour over the following week. Another strength was our choice to measure participants' *perceptions* of their identity‐relevant behaviour. Perceptions of identity‐behaviour consistency may be drawn from an individual's actual behaviour, the responses of other people to their behaviour, or from combinations of these stimuli (Burke & Stets, 2009). Because perceptions of identity‐behaviour consistency are generated through a process of individual meaning‐making, they may not be fully congruent with an identity holder's actual behaviour. Therefore, these perceptions may be more theoretically relevant to the identity verification process compared to actual behaviours (Burke & Stets, 2009; Cast & Burke, 2002). While our approach has theoretical strengths, we acknowledge the importance of exploring actual engagement in identity‐relevant behaviours. The relationship between endorsing an identity and engaging in identity‐relevant behaviours is particularly important in the context of exercise identities, given the health benefits of exercise. Therefore, future researchers should consider repeating the methods used in this study while measuring exercise behaviour as the primary outcome.

Participant recruitment was a strength of this study; participants from crowdsourcing platforms such as Prolific provide reliable data that is comparable to conventional sampling methods (e.g., ‘in‐lab’ samples; Du et al., 2024; Lutz, 2016) with the added benefit of drawing from an international sample (Prolific, 2023). Limitations of recruiting through prolific include careless responding; however, we included attention and comprehension checks throughout each survey, which all participants passed. Other limitations include a short follow‐up; participants may have been unable to overcome the barriers causing their identity‐behaviour discrepancy within this time. We also see advantages to a one‐week follow‐up. We reason that people plan for exercise in one‐week intervals. Future research could use ecological momentary assessment to replicate our findings across a longer time interval. A further study strength is that we controlled for trait guilt and shame in our analyses which is recommended (Tangney, 2003). We also controlled for age and gender. Researchers should consider whether age or gender influences how self‐compassion impacts people's affective responses to identity‐behaviour discrepancy.

## CONCLUSION

This study sought to explain inconsistent findings about, and qualify the role of, negative affect within identity theory through examining self‐compassion as a moderator. We did not find support for identity theory's propositions that negative affect promotes the regulation of identity‐consistent behaviour after an identity‐inconsistent situation. Self‐compassion moderated the relationship between guilt and perceptions of identity‐behaviour consistency after an identity‐inconsistent situation. Self‐compassion may play a role in how people respond to and regulate their behaviour in the face of identity‐inconsistent situations, and the propositions of the identity control system may hold true among people who are more self‐compassionate.

## AUTHOR CONTRIBUTIONS

**Shaelyn M. Strachan:** Conceptualization; writing – original draft; methodology; supervision; investigation; project administration; writing – review and editing; funding acquisition; resources. **Sasha M. Kullman:** Data curation; formal analysis; writing – original draft; visualization; investigation; writing – review and editing. **Marko Dobrovolskyi:** Writing – original draft; writing – review and editing. **Vianney Z. Vega:** Writing – original draft; writing – review and editing. **Alexandra Yarema:** Investigation. **Caity Patson:** Investigation.

## CONFLICT OF INTEREST STATEMENT

The authors declare no conflict of interest.

## Supporting information


Data S1

## Data Availability

In line with the approved ethical protocol for this study, data is not publicly archived. Data is available upon request by contacting the corresponding author.

## Footnotes

^1^
Other researchers have created the variables of guilt‐free shame and shame‐free guilt using residual analysis (e.g., Sabiston et al., 2010). This approach has been called into question (Sabini & Silver, 1997). Given the drawbacks of residual analysis (Darlington & Smulders, 2001), we report on guilt and shame without modification. We ran exploratory analyses using guilt‐free shame and shame‐free guilt and found no difference in our results.
