# Supplementary material for: Explaining the self‐regulatory role of affect in identity theory: The role of self‐compassion
Source: Br J Health Psychol. 2025 Feb 1;30(1):e12783. doi: 10.1111/bjhp.12783 (PMC11786238; doi:10.1111/bjhp.12783)
Supplement: Supplementary file 1 — Data S1 [file BJHP-30-0-s001.docx]

| **Supplemental Table 1**  *Participant Descriptive Information* | | |
| --- | --- | --- |
| Variable | *Mean / Frequency* | *SD / Percent* |
| Age | 32.5 | 10.8 |
| Education |  |  |
| Some high-school | 3 | 1.1 % |
| Highschool | 39 | 14.1 % |
| Some college or university | 40 | 14.4 % |
| A college degree | 28 | 10.1 % |
| An undergraduate university degree | 123 | 44.4 % |
| A master’s degree | 42 | 15.2 % |
| A doctorate | 2 | 0.7 % |
| Geographic location |  |  |
| Australia | 3 | 1.1 % |
| Austria | 1 | .4 % |
| Canada | 13 | 4.7 % |
| Chile | 2 | .7 % |
| Czech Republic | 3 | 1.1 % |
| Estonia | 1 | .4 % |
| Finland | 3 | 1.1 % |
| France | 1 | .4 % |
| Germany | 5 | 1.8 % |
| Greece | 12 | 4.3 % |
| Hungary | 1 | .4 % |
| Ireland | 3 | 1.1 % |
| Israel | 2 | .7 % |
| Italy | 12 | 4.3 % |
| Mexico | 19 | 6.9 % |
| Netherlands | 8 | 2.9 % |
| New Zealand | 1 | .4 % |
| Norway | 1 | .4 % |
| Poland | 21 | 7.6 % |
| Portugal | 21 | 7.6 % |
| Slovenia | 2 | .7 % |
| South Africa | 20 | 7.2 % |
| Spain | 9 | 3.2 % |
| Sweden | 3 | 1.1 % |
| United Kingdom | 89 | 32.1 % |
| United States | 21 | 7.6 % |
| Ethnicity |  |  |
| Black (e.g., African, American, Canadian, Caribbean) | 46 | 16.6 % |
| Chinese | 3 | 1.1 % |
| Filipino | 1 | .4 % |
| Korean | 1 | .4 % |
| Non-White Latin American (including indigenous person from Central and South America) | 13 | 4.7 % |
| Non-White North African (e.g., Egyptian, Libyan) | 1 | .4 % |
| Person of Mixed Origin | 13 | 4.7 % |
| South African | 2 | .7 % |
| South African | 1 | .4 % |
| South Asian (e.g., Bangladeshi, Pakistani, Indian from India, Trinidadian, Sri Lankan) | 7 | 2.5 % |
| South East Asian (e.g., Myanma, Cambodian, Laotian, Malaysian, Thai, Vietnamese, Indonesian) | 2 | .7 % |
| White (of European descent) | 183 | 66.1 % |
| Other | 4 | 1.4 % |
| Gender |  |  |
| Man | 136 | 49.1 % |
| Woman | 139 | 50.2 % |
| Queer | 1 | .4 % |
| Non-Binary | 1 | .4 % |
| Marital status |  |  |
| Single | 152 | 54.9 % |
| Common-law | 44 | 15.9 % |
| Married | 75 | 27.1 % |
| Separated | 3 | 1.1 % |
| Divorced | 3 | 1.1 % |

| **Supplemental Table 2**  *Descriptive Statistics and Scale Reliability* | | | | | | | |
| --- | --- | --- | --- | --- | --- | --- | --- |
| Variable | N | Mean | SD | Range | Minimum | Maximum | Alpha |
| Exercise Identity | 276 | 5.71 | 0.78 | 1 to 7 | 3.22 | 7.00 | .866 |
| Self-compassion | 263 | 2.97 | 0.64 | 1 to 5 | 1.12 | 4.79 | .922 |
| State shame | 274 | 11.93 | 2.60 | 1 to 5 | 6.00 | 20.00 | .861 |
| State guilt | 275 | 11.03 | 2.49 | 1 to 5 | 5.00 | 19.00 | .790 |
| Trait shame | 275 | 50.39 | 10.51 | 16 to 80 | 18.00 | 74.00 | .818 |
| Trait guilt | 276 | 64.86 | 7.28 | 16 to 80 | 41.00 | 80.00 | .738 |
| Intentions to increase PA | 277 | 6.03 | 1.16 | 1 to 7 | 1.00 | 7.00 | NA |
| T1 weekly minutes of mild PA | 275 | 91.99 | 101.58 | NA | 0 | 320.00 | NA |
| T1 weekly minutes of moderate PA | 274 | 90.13 | 95.57 | NA | 0 | 400.00 | NA |
| T1 weekly minutes of vigorous PA | 276 | 73.20 | 82.20 | NA | 0 | 420.00 | NA |
| T1 identity-consistent perceptions | 277 | 50.12 | 21.92 | 0 to 100 | 0 | 100 | NA |
| T2 weekly minutes of mild PA | 276 | 102.99 | 119.48 | NA | 0 | 480.00 | NA |
| T2 weekly minutes of moderate PA | 276 | 115.12 | 112.24 | NA | 0 | 480.00 | NA |
| T2 weekly minutes of vigorous PA | 276 | 107.07 | 106.39 | NA | 0 | 450.00 | NA |
| T2 identity-consistent perceptions | 277 | 59.39 | 26.95 | 0 to 100 | 0.00 | 100.00 | NA |
| T1 = Time 1  T2 = Time 2 | | | | | | | |

| **Supplemental Table 3**  *Association between state shame and intentions to increase physical activity* | | | | | |
| --- | --- | --- | --- | --- | --- |
| Variable | *b* | 95% CI for *b* | SE of b | β | *p* |
| Constant | 5.560 | 3.080 to 8.040 | 1.260 |  | <.001 |
| Age | .003 | -.010 to .017 | .007 | .030 | .638 |
| Gender (Man) | .020 | -2.295 to 2.334 | 1.176 | .008 | .987 |
| Gender (Woman) | .125 | -2.197 to 2.446 | 1.179 | .054 | .916 |
| Trait Shame | -.006 | -.021 to .008 | .007 | -.057 | .379 |
| State Shame | .052 | -.005 to .109 | .029 | .115 | .075 |

| **Supplemental Table 4**  *Association between state shame and Time 2 identity-consistent perceptions* | | | | | |
| --- | --- | --- | --- | --- | --- |
| Variable | *b* | 95% CI for *b* | SE of b | β | *p* |
| Constant | 24.289 | -32.694 to 81.271 | 28.941 |  | .402 |
| Age | .279 | -.036 to .594 | .160 | .110 | .082 |
| Gender (Man) | 37.047 | -16.137 to 90.231 | 27.011 | .685 | .171 |
| Gender (Woman) | 32.147 | -21.198 to. 85.492 | 27.093 | .594 | .236 |
| Trait Shame | -.259 | -.587 to .069 | .167 | -.100 | .122 |
| State Shame | .387 | -.921 to 1.695 | .664 | .037 | .561 |

| **Supplemental Table 5**  *Association between state guilt and intentions to increase physical activity* | | | | | |
| --- | --- | --- | --- | --- | --- |
| Variable | *b* | 95% CI for *b* | SE of b | β | *p* |
| Constant | 4.200 | 1.496 to 6.904 | 1.373 |  | .002 |
| Age | .003 | -.010 to .016 | .007 | .030 | .635 |
| Gender (Man) | -.003 | -2.301 to 2.295 | 1.167 | -.001 | .998 |
| Gender (Woman) | -.044 | -2.345 to 2.257 | 1.169 | -.019 | .970 |
| Trait Guilt | .018 | -.002 to .038 | .010 | .113 | .070 |
| State Guilt | .052 | -.005 to .109 | .029 | .112 | .073 |

| **Supplemental Table 6**  *Association between state guilt and Time 2 identity-consistent perceptions* | | | | | |
| --- | --- | --- | --- | --- | --- |
| Variable | *b* | 95% CI for *b* | SE of b | β | *p* |
| Constant | 22.976 | -39.618 to -85.571 | 31.792 |  | .470 |
| Age | .254 | -.053 to .560 | .156 | .101 | .104 |
| Gender (Man) | 35.432 | -17.761 to 88.624 | 27.017 | .658 | .191 |
| Gender (Woman) | 29.141 | -24.124 to 82.406 | 27.053 | .541 | .282 |
| Trait Guilt | -.038 | -.491 to .415 | .230 | -.010 | .869 |
| State Guilt | -.124 | -1.443 to 1.195 | .670 | -.011 | .854 |

| **Supplemental Table 7**  *Interaction effect of self-compassion and state guilt on exercise intentions* | | | | | |
| --- | --- | --- | --- | --- | --- |
| Variable | *b* | 95% CI for *b* | SE of b | t | *p* |
| Constant | 4.7215 | 2.1205 to 7.3225 | 1.3207 | 3.5751 | .0004 |
| State Guilt | .0529 | -.0051 to .1109 | .0294 | 1.7972 | .0735 |
| Self-Compassion | .0984 | -.1305 to .3273 | .1162 | .8467 | .3979 |
| State Guilt * Self-Compassion | -.0276 | -.1124 to .0573 | .0431 | -.6400 | .5228 |
| Trait Guilt | .0202 | .0000 to .0404 | .0103 | 1.9679 | .0502 |
| Age | .0006 | -.0131 to .0143 | .0070 | .0857 | .9318 |
| Gender (Man) | -.0480 | -2.3321 to 2.2361 | 1.1598 | -.0414 | .9670 |
| Gender (Woman) | -.0077 | -2.2942 to 2.2788 | 1.1610 | -.0067 | .9947 |

| **Supplemental Table 8**  *Interaction effect of self-compassion and state shame on exercise intentions* | | | | | |
| --- | --- | --- | --- | --- | --- |
| Variable | *b* | 95% CI for *b* | SE of b | t | *p* |
| Constant | 6.3138 | 4.5220 to 8.1056 | .9098 | 6.9400 | .0000 |
| State Shame | .0409 | -.0168 to .0985 | .0293 | 1.3969 | .1637 |
| Self-Compassion | .1238 | -.1378 to.3855 | .1328 | .9323 | .3521 |
| State Shame * Self-Compassion | -.0271 | -.1147 to .0605 | .0445 | -.6086 | .5433 |
| Trait Shame | .0032 | -.0139 to .0203 | .0087 | .3711 | .7109 |
| Age | .0004 | -.0136 to .0144 | .0071 | .0582 | .9537 |
| Gender (Man) | -.5277 | -2.1724 to 1.1170 | .8351 | -.6319 | .5280 |
| Gender (Woman) | -.3908 | -2.0465 to 1.2648 | .8406 | -.4649 | .6424 |

| **Supplemental Table 9**  *Interaction effect of self-compassion and state shame on Time 2 identity-consistent perceptions* | | | | | |
| --- | --- | --- | --- | --- | --- |
| Variable | *b* | 95% CI for *b* | SE of b | t | *p* |
| Constant | 45.7134 | 4.9429 to 86.4839 | 20.7013 | 2.2082 | .0281 |
| State Shame | .6085 | -.7029 to 1.9200 | .6659 | .9138 | .3617 |
| Self-Compassion | 5.7559 | -.1971 to 11.7089 | 3.0227 | 1.9043 | .0580 |
| State Shame * Self-Compassion | 1.1660 | -.8271 to 3.1591 | 1.0120 | 1.1522 | .2503 |
| Trait Shame | -.1125 | -.5009 to .2759 | .1972 | -.5703 | .5690 |
| Age | .2929 | -.0254 to .6113 | .1616 | 1.8123 | .0711 |
| Gender (Man) | 12.7824 | -24.6413 to 50.2061 | 19.0020 | .6727 | .5018 |
| Gender (Woman) | 7.4243 | -30.2483 to 45.0970 | 19.1284 | .3881 | .6982 |
